# Supplementary material for: Modeling glioblastoma heterogeneity as a dynamic network of cell states
Source: Mol Syst Biol. 2021 Sep 16;17(9):e10105. doi: 10.15252/msb.202010105 (PMC8444284; doi:10.15252/msb.202010105)
Supplement: Supplementary file 5 — Source Data for Figure 3 [file MSB-17-e10105-s001.zip › Figure3A_sourcedata/GSEA_3065/nosigdb_state3.GseaPreranked.1622718426028/LARSSON_STATE_3.html]

Details for gene set LARSSON\_STATE\_3[GSEA]

|  || Dataset | state3 |
| Phenotype | NoPhenotypeAvailable |
| Upregulated in class | na\_pos |
| GeneSet | LARSSON\_STATE\_3 |
| Enrichment Score (ES) | 0.9982691 |
| Normalized Enrichment Score (NES) | 3.876227 |
| Nominal p-value | 0.0 |
| FDR q-value | 0.0 |
| FWER p-Value | 0.0 |
Table: GSEA Results Summary

  

Fig 1: Enrichment plot: LARSSON\_STATE\_3      
 Profile of the Running ES Score & Positions of GeneSet Members on the Rank Ordered List

  

| PROBE | GENE SYMBOL | GENE\_TITLE | RANK IN GENE LIST | RANK METRIC SCORE | RUNNING ES | CORE ENRICHMENT || 1 | BCAN |  |  | 0 | 0.883 | 0.0236 | Yes |
| 2 | VGF |  |  | 1 | 0.745 | 0.0436 | Yes |
| 3 | SCRG1 |  |  | 3 | 0.645 | 0.0608 | Yes |
| 4 | CENPF |  |  | 4 | 0.619 | 0.0774 | Yes |
| 5 | PRKDC |  |  | 6 | 0.588 | 0.0930 | Yes |
| 6 | HSP90B1 |  |  | 7 | 0.563 | 0.1081 | Yes |
| 7 | PDIA6 |  |  | 8 | 0.549 | 0.1228 | Yes |
| 8 | B2M |  |  | 11 | 0.538 | 0.1370 | Yes |
| 9 | SPCS2 |  |  | 12 | 0.532 | 0.1512 | Yes |
| 10 | MTRNR2L12 |  |  | 13 | 0.532 | 0.1655 | Yes |
| 11 | PTPRZ1 |  |  | 14 | 0.532 | 0.1797 | Yes |
| 12 | SYNE2 |  |  | 16 | 0.529 | 0.1938 | Yes |
| 13 | PLAT |  |  | 18 | 0.513 | 0.2074 | Yes |
| 14 | APLP2 |  |  | 20 | 0.506 | 0.2208 | Yes |
| 15 | LMAN1 |  |  | 21 | 0.496 | 0.2341 | Yes |
| 16 | PTN |  |  | 22 | 0.493 | 0.2473 | Yes |
| 17 | POSTN |  |  | 24 | 0.487 | 0.2603 | Yes |
| 18 | ASPM |  |  | 25 | 0.480 | 0.2731 | Yes |
| 19 | CD63 |  |  | 26 | 0.480 | 0.2860 | Yes |
| 20 | CANX |  |  | 27 | 0.479 | 0.2988 | Yes |
| 21 | PGRMC1 |  |  | 29 | 0.473 | 0.3114 | Yes |
| 22 | CALR |  |  | 30 | 0.473 | 0.3240 | Yes |
| 23 | LAPTM4B |  |  | 31 | 0.473 | 0.3367 | Yes |
| 24 | CD9 |  |  | 32 | 0.473 | 0.3494 | Yes |
| 25 | TIMP1 |  |  | 35 | 0.470 | 0.3617 | Yes |
| 26 | ABHD2 |  |  | 36 | 0.467 | 0.3742 | Yes |
| 27 | GPM6A |  |  | 37 | 0.460 | 0.3865 | Yes |
| 28 | GRIA2 |  |  | 38 | 0.460 | 0.3988 | Yes |
| 29 | SQLE |  |  | 39 | 0.457 | 0.4111 | Yes |
| 30 | PRDX4 |  |  | 41 | 0.455 | 0.4232 | Yes |
| 31 | TOP2A |  |  | 42 | 0.448 | 0.4352 | Yes |
| 32 | HSPA5 |  |  | 43 | 0.447 | 0.4471 | Yes |
| 33 | ALCAM |  |  | 45 | 0.440 | 0.4588 | Yes |
| 34 | GGH |  |  | 46 | 0.439 | 0.4706 | Yes |
| 35 | DYNC1H1 |  |  | 47 | 0.437 | 0.4823 | Yes |
| 36 | CALU |  |  | 48 | 0.437 | 0.4940 | Yes |
| 37 | APP |  |  | 49 | 0.437 | 0.5057 | Yes |
| 38 | RTN3 |  |  | 50 | 0.435 | 0.5173 | Yes |
| 39 | FGFBP3 |  |  | 51 | 0.431 | 0.5289 | Yes |
| 40 | NCAM1 |  |  | 52 | 0.430 | 0.5404 | Yes |
| 41 | CCDC47 |  |  | 54 | 0.424 | 0.5516 | Yes |
| 42 | PDIA3 |  |  | 55 | 0.419 | 0.5628 | Yes |
| 43 | MALAT1 |  |  | 57 | 0.412 | 0.5738 | Yes |
| 44 | WLS |  |  | 58 | 0.407 | 0.5847 | Yes |
| 45 | MKI67 |  |  | 59 | 0.407 | 0.5956 | Yes |
| 46 | HSP90AA1 |  |  | 60 | 0.402 | 0.6063 | Yes |
| 47 | SPTBN1 |  |  | 61 | 0.394 | 0.6169 | Yes |
| 48 | DSEL |  |  | 62 | 0.392 | 0.6274 | Yes |
| 49 | PON2 |  |  | 63 | 0.389 | 0.6378 | Yes |
| 50 | PIF1 |  |  | 64 | 0.389 | 0.6482 | Yes |
| 51 | TFPI2 |  |  | 65 | 0.388 | 0.6586 | Yes |
| 52 | STT3B |  |  | 66 | 0.384 | 0.6689 | Yes |
| 53 | GPNMB |  |  | 67 | 0.383 | 0.6792 | Yes |
| 54 | LPL |  |  | 68 | 0.381 | 0.6893 | Yes |
| 55 | ATP6AP2 |  |  | 69 | 0.380 | 0.6995 | Yes |
| 56 | MANF |  |  | 70 | 0.376 | 0.7096 | Yes |
| 57 | HNRNPU |  |  | 71 | 0.373 | 0.7196 | Yes |
| 58 | EMC7 |  |  | 72 | 0.372 | 0.7295 | Yes |
| 59 | TMBIM6 |  |  | 73 | 0.372 | 0.7395 | Yes |
| 60 | HSP90AB1 |  |  | 74 | 0.371 | 0.7494 | Yes |
| 61 | TMED9 |  |  | 75 | 0.370 | 0.7593 | Yes |
| 62 | SPCS3 |  |  | 76 | 0.370 | 0.7692 | Yes |
| 63 | HMGCR |  |  | 77 | 0.369 | 0.7791 | Yes |
| 64 | SSR4 |  |  | 78 | 0.368 | 0.7890 | Yes |
| 65 | MYH10 |  |  | 79 | 0.366 | 0.7988 | Yes |
| 66 | DST |  |  | 80 | 0.365 | 0.8086 | Yes |
| 67 | LRPAP1 |  |  | 81 | 0.361 | 0.8182 | Yes |
| 68 | ITM2B |  |  | 82 | 0.361 | 0.8279 | Yes |
| 69 | SLC44A1 |  |  | 83 | 0.361 | 0.8376 | Yes |
| 70 | CD320 |  |  | 84 | 0.361 | 0.8472 | Yes |
| 71 | NASP |  |  | 85 | 0.360 | 0.8569 | Yes |
| 72 | IQGAP1 |  |  | 86 | 0.359 | 0.8665 | Yes |
| 73 | FUS |  |  | 87 | 0.358 | 0.8761 | Yes |
| 74 | SOX6 |  |  | 88 | 0.358 | 0.8857 | Yes |
| 75 | HACD3 |  |  | 89 | 0.356 | 0.8952 | Yes |
| 76 | GPC1 |  |  | 90 | 0.355 | 0.9047 | Yes |
| 77 | EMP3 |  |  | 91 | 0.354 | 0.9142 | Yes |
| 78 | MDM4 |  |  | 92 | 0.351 | 0.9236 | Yes |
| 79 | TM9SF3 |  |  | 93 | 0.351 | 0.9330 | Yes |
| 80 | MMP16 |  |  | 94 | 0.351 | 0.9424 | Yes |
| 81 | FAT1 |  |  | 95 | 0.350 | 0.9518 | Yes |
| 82 | LAMC1 |  |  | 96 | 0.349 | 0.9611 | Yes |
| 83 | PLD5 |  |  | 97 | 0.348 | 0.9704 | Yes |
| 84 | BSG |  |  | 98 | 0.347 | 0.9797 | Yes |
| 85 | KCNQ1OT1 |  |  | 99 | 0.347 | 0.9890 | Yes |
| 86 | RPN2 |  |  | 100 | 0.345 | 0.9983 | Yes |
Table: GSEA details [plain text format]

  

Fig 2: LARSSON\_STATE\_3: Random ES distribution      
 Gene set null distribution of ES for **LARSSON\_STATE\_3**
